# Pathogenic Mechanisms of Bicuspid Aortic Valve Aortopathy

**DOI:** 10.3389/fphys.2017.00687

**Published:** 2017-09-25

**Authors:** Noor M. Yassine, Jasmine T. Shahram, Simon C. Body

**Affiliations:** Department of Anesthesiology, Perioperative and Pain Medicine, Brigham and Women's Hospital Boston, MA, United States

**Keywords:** bicuspid aortic valve, genetics, thoracic aortic aneurysm, fibrillin, GATA4, transforming growth factor-β

## Abstract

Bicuspid aortic valve (BAV) is the most common congenital valvular defect and is associated with ascending aortic dilation (AAD) in a quarter of patients. AAD has been ascribed both to the hemodynamic consequences of normally functioning and abnormal BAV morphology, and to the effect of rare and common genetic variation upon function of the ascending aortic media. AAD manifests in two overall and sometimes overlapping phenotypes: that of aortic root aneurysm, similar to the AAD of Marfan syndrome; and that of tubular AAD, similar to the AAD seen with tricuspid aortic valves (TAVs). These aortic phenotypes appear to be independent of BAV phenotype, have different embryologic origins and have unique etiologic factors, notably, regarding the role of hemodynamic changes inherent to the BAV phenotype. Further, in contrast to Marfan syndrome, the AAD seen with BAV is infrequently present as a strongly inherited syndromic phenotype; rather, it appears to be a less-penetrant, milder phenotype. Both reduced levels of normally functioning transcriptional proteins and structurally abnormal proteins have been observed in aneurysmal aortic media. We provide evidence that aortic root AAD has a stronger genetic etiology, sometimes related to identified common non-coding fibrillin-1 (*FBN1*) variants and other aortic wall protein variants in patients with BAV. In patients with BAV having tubular AAD, we propose a stronger hemodynamic influence, but with pathology still based on a functional deficit of the aortic media, of genetic or epigenetic etiology. Although it is an attractive hypothesis to ascribe common mechanisms to BAV and AAD, thus far the genetic etiologies of AAD have not been associated to the genetic etiologies of BAV, notably, not including BAV variants in *NOTCH1* and *GATA4*.

## Introduction

Bicuspid aortic valve (BAV) disease is the most common congenital valvular abnormality, with an incidence in male Caucasians of ~1.5% and lower incidence in female and non-Caucasian individuals (Michelena et al., 2014). A spectrum of other infrequent congenital abnormalities, such as coarctation of the aorta, has been described with BAV (Michelena et al., 2014; Prakash et al., 2014). There is moderate heritability of BAV, but the vast majority of affected patients do not possess other syndromic features and have indeterminate inheritance (Prakash et al., 2014). Unlike the majority of congenital cardiac disease, BAV is most frequently diagnosed in adulthood, notably, with the onset of aortic valvular dysfunction, ascending aortic dilation (AAD; Michelena et al., 2008, 2011; McKellar et al., 2010) or endocarditis (Kiyota et al., 2017). More than 50%, and perhaps as high as 75%, of patients with BAV undergo aortic valve replacement during their lifetime (Michelena et al., 2011). Similarly, more than 25% of patients with BAV undergo aortic surgery, often concurrent with aortic valve replacement (Michelena et al., 2011), with most aortic surgery performed for dilation of the aortic root or ascending aorta, and rarely for aortic dissection.

## Clinical phenotyping of BAV-related aortopathy

**Figure 1:**
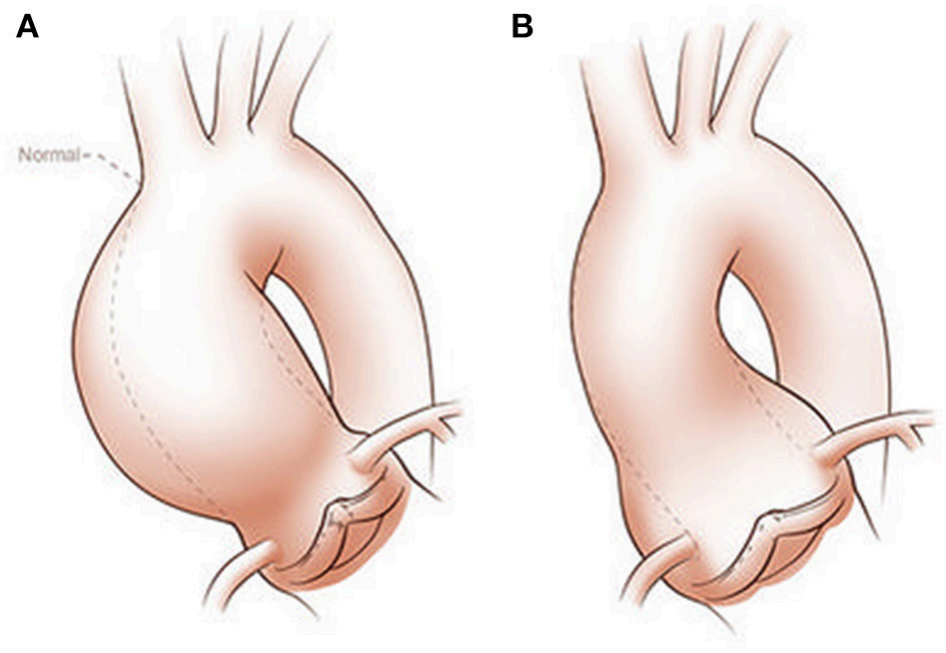
Classification of ascending aortic dilation phenotypes. Ascending aortic dilations (aneurysms) can be classified into a tubular phenotype **(A)** located above the sinotubular junction (STJ) and an aortic root phenotype **(B)** located below the STJ. This classification is neither explanatory nor complete, as ascending aortic dilations frequently extend above or below the STJ and may extend into the aortic arch or beyond. However, it provides a functional distinction based on embryogenic origins of the aorta and surgical approaches. Copyright: Glen Oomen, M.Sc. (http://www.glenoomen.com/medical-illustration/b2mswldypmppf644fjbm1k9kc3w472)

BAV aortopathy is not a single clinical phenotype (Cotrufo and Della Corte, 2009; Della Corte and Bancone, 2012; Della Corte, 2014; Della Corte et al., 2014a,b). There is marked variability of AAD dimensional phenotypes with aortic dilation observed heterogeneously from the aortic annulus to the aortic arch (Girdauskas and Borger, 2013; Della Corte, 2014). Although several classification systems for AAD exist, the simplest divides the spectrum into two classes: aortic root AAD vs. tubular AAD (Figure 1). Although simplistic, it provides an attractive distinction that can be used for initial work and is perhaps justified based on embryonic origin of the tissue and functional characterization of valve morphology and disease (Della Corte et al., 2007). Evidence supporting this classification comes from three sources: longitudinal follow-up of patients with BAV and AAD notably after aortic valve replacement; imaging of aortic blood flow to define areas of increased wall stress; and histologic studies that are consistent with the natural history of the disease and imaging findings.

### Tubular ascending aortic phenotype

The most frequent clinical presentation of BAV is a murmur or incidental finding of calcific aortic valve disease (CAVD) after the age of 50 years, occurring on average 15 years before symptoms of tricuspid aortic valve (TAV)-related aortic stenosis typically occur. Commonly, there is a concurrent AAD above the sinotubular junction with high-velocity and turbulent flow eccentricity from the stenotic BAV, toward the convex aortic surface. This eccentricity has been described to cause altered shear stresses from increased velocities through the stenotic valve (Della Corte et al., 2007), but it is apparent that abnormal flow patterns exist in the absence of stenosis, merely from the presence of the bicuspid valve (Entezari et al., 2014). Fusion of the right and non-coronary cusps has been more frequently associated with dilation of the tubular ascending aorta, but not exclusively so (Della Corte et al., 2014a; Girdauskas et al., 2016b). However, it is not clear that hemodynamic forces are the only etiologic factor. The supporting clinical data tend to be anecdotal, including a low incidence of aortic events such as surgery or AAD after resection of the stenotic aortic valve (Girdauskas et al., 2012, 2014). However, dilation of the aorta continues after curative aortic valve replacement (Girdauskas et al., 2016a; Regeer et al., 2016; Naito et al., 2017). To date, there is no valid prediction index for aortic dilation (Abdulkareem et al., 2013; Geisbusch et al., 2014; Gagne-Loranger et al., 2016).

Imaging data are more robust and support a hemodynamic component to tubular AAD. Using magnetic resonance imaging to measure 3-dimensional blood flow through the valve and aorta over time creates 4-dimensional maps of blood flow within the aorta. This technology provides the most detailed hemodynamic assessment and should be integrated into future investigation of the aortic wall cell biology. But phenotypic imaging still has significant issues of measurement classification and of comparison of data from different imaging modalities (Park et al., 2017), between pediatric and adult clinical imaging methods, and across the spectra of body habitus and age. These issues have previously limited our ability to identify biological mechanisms of AAD across a wide phenotypic spectrum such as root aneurysm vs. ascending aortic aneurysm, and dilated vs. normal aorta. Marked improvements in genetics, genomics, epigenetics and molecular biology over the last two decades have overcome limitations in imaging phenotypes, enabling phenotyping based instead on the disease biology.

### Aortic root dilation phenotype

About 15% of patients with AAD have a dilated aortic root characterized by dilated sinuses and annulus, often including the sinotubular junction; these patients present at a younger age because of the severity of aortic incompetence, occurring in the absence of CAVD. This phenotype has been more frequently associated with right-left cusp fusion (Jassal et al., 2010; Della Corte et al., 2014a), but not reliably so (Girdauskas et al., 2016b; Habchi et al., 2017). In contrast to the accelerated CAVD seen in patients with BAV having the tubular ascending aortic phenotype, which perhaps results from abnormal aortic shear stress, this phenotype probably results from a primary structural lesion of the aortic root and annulus rather than occurring secondarily to altered aortic wall stresses, with the aortic incompetence directly resulting from the aortic annulus and root lesion.

This simplistic classification of AAD emphasizes limitations in our understanding. Some patients with a seemingly normally functioning BAV exhibit early and marked tubular AAD, while conversely, many patients with long-standing BAV and CAVD do not have AAD. The association between BAV morphotype (right-left vs. right-non-coronary fusion) and aortic phenotype (root vs. tubular) is weak (Habchi et al., 2017), and mechanistic studies still do not explain the overall phenotypes. It is recognized that merely accounting for the severity of valve disease, the configuration of the valve, the presence or absence of a raphe, or aortic dimensions does not describe the severity of aortic wall disease, or risk of future adverse events (Fedak et al., 2016). More likely, approaches that make use of molecular markers of aortic wall dysfunction, specific imaging phenotypes using 4-dimensional flow MRI and genetic risk factors will have greater precision.

## Embryology of the aortic root and ascending aorta

The two broad root and tubular phenotypes of AAD, and their dissimilarity to descending thoracic aortic disease, reflect the embryonic origin of aortic cell lineage. These have been recently reviewed (Martin et al., 2015; Anderson et al., 2016). The aortic root and ascending aortic phenotypes match embryologic dissemination of ectodermal and mesenchymal cells from the cardiac neural crest (CNC) and second heart field (SHF), respectively, into the truncal outflow tract (Jiang et al., 2000).

**Figure 2:**
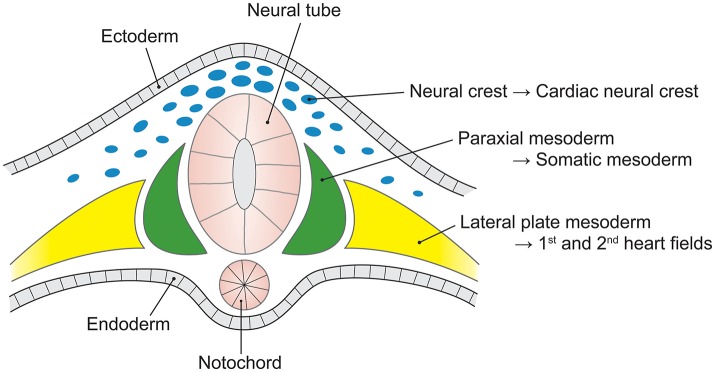
The embryological origins of the thoracic vasculature and outflow tract. The embryonic first and second heart fields are derived from the lateral plate mesoderm. The first heart field forms the early heart tube, into which second heart field cells migrate to form the convoluting heart. The cardiac neural crest is derived from a clone of neural crest cells that migrate along the third and fourth pharyngeal arches to form the head and upper limb arteries along with the ascending aorta and aortic arch. The pulmonary artery is formed from neural crest cells that migrate along the sixth pharyngeal arch.

The embryonic heart and aorta are developed from three precursor cell populations: proepicardial cells, cardiogenic mesodermal cells, and CNC cells. Proepicardial progenitors principally form the epicardium and coronary vessels (Figure 2). Cardiogenic mesodermal cells contribute to the first and second heart fields. The first heart field (created first in embryogenesis) forms the early embryonic heart tube that contributes to the left ventricle and portions of the right ventricle and atria. The SHF (created second in embryogenesis) is the primary source of the outflow tract (conus cordis and truncus arteriosus) as well as the majority of the right ventricle and the venous pole of the heart (Dyer and Kirby, 2009). The SHF contributes both myocardium to the outflow tract and smooth muscle to the truncus arteriosus. CNC cells arise from the ectodermal dorsal neural tube and migrate through the pharyngeal arches to the anterior domain of the SHF. CNC mesenchymal cells contribute to the aortopulmonary valves and outflow tracts, ascending aorta and arch, and proximal pulmonary artery (Snarr et al., 2008; Plein et al., 2015; Jiao et al., 2016).

**Figure 3:**
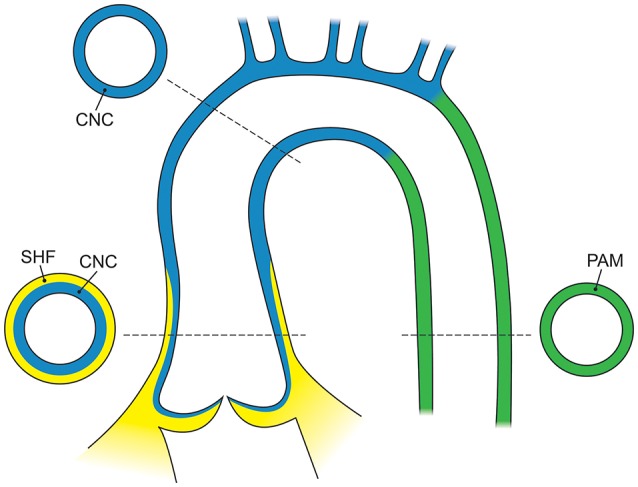
The embryological origins of smooth muscle cells of the aorta. The adult aortic root is principally derived from the second heart field (yellow), whereas the ascending aorta and arch are derived from the cardiac neural crest (blue) and the descending aorta is derived from the paraxial mesoderm (green). In the adult aorta, smooth muscle cells of both the second heart field and neural crest form the aortic root and ascending aorta, while the descending aorta below the ductus arteriosus is composed of cells from the lateral mesoderm. CNC, cardiac neural crest; SHF, second heart field; PAM, paraxial mesoderm.

**Figure 4:**
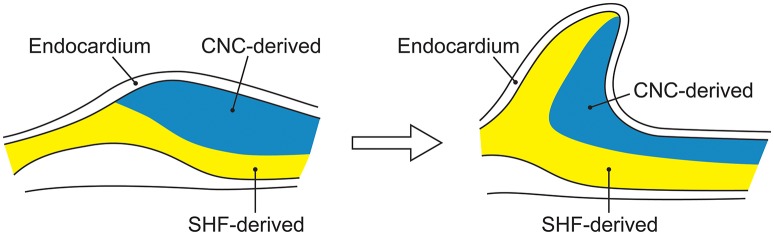
The embryological origins of smooth muscle cells of the aortic valve. Embryonic development of the aorta valve incorporates smooth muscle cells derived from both the second heart field and cardiac neural crest. Although, portrayed as two layers of distinct cellular origins with cardiac neural crest-derived cells occupying the fibrosal side (left side of each figure) of the valve and second heart field-derived cells occupying the ventricular side of the valve (left side of each figure), there is evidence for endocardial-to-mesenchymal transformation in the endocardial cushions that develop into the valve, along with considerable plasticity of all elements of the developing valve.

**Figure 5:**
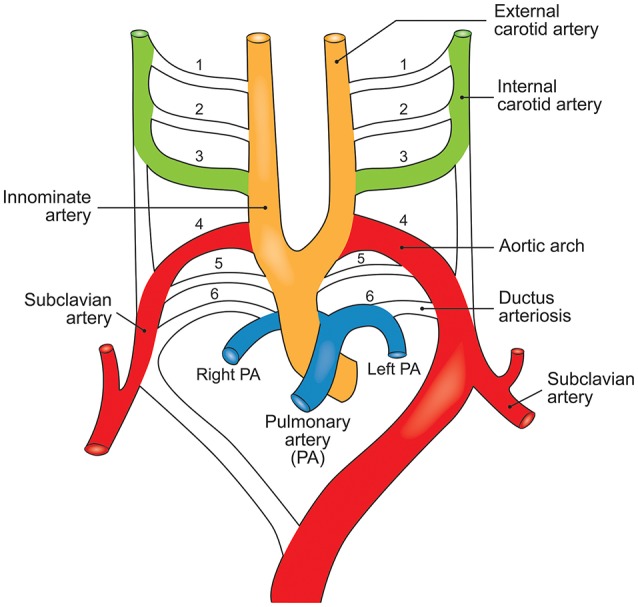
The embryological destinations of the intrathoracic vasculature. Development of the upper body arterial tree is predicated on expansion, migration, and apoptosis of cell populations in the branchial arteries to yield its neonatal structure. The branchial arteries are numbered 1–6.

SHF and CNC cells are not randomly intermixed in the outflow tract and ascending aorta. Cell lineage tracking, principally in mouse embryos, reveals that SHF mesenchymal cells are dominantly located in the aortic root. Above the aortic root, SHF cells are more localized to the adventitial side of the aortic media until they are no longer present in the developing structures (Waldo et al., 2005; Harmon and Nakano, 2013). In contrast, CNC cells populate the intimal edge of the ascending aorta, occupying the whole media as far as the left subclavian artery (Pfaltzgraff et al., 2014; Figure 3). The transition from SHF to CNC predominance occurs closer to the root on the dorsal (posterior) side of the aorta than on the ventral (anterior) side (Sawada et al., 2017). Yet, CNC cells are required for embryonic development of the aortic and pulmonary valves, and for outflow tract development and septation (Phillips et al., 2013). Although debated, in the developing aortic valve CNC cells populate the aortic surface (fibrosa) of the valve, while SHF cells populate the ventricular surface (ventricularis) of the valve (Figure 4). The fibrosa has a high type I and III collagen content arranged in a concentric fashion and is notable for its propensity to develop CAVD, while the ventricularis is notable for its high elastin content arranged in a radial fashion and is spared from development of CAVD. These histologic differences that have a vital functional role in aortic valve biomechanics may reflect embryologic origins from SHF and CNC, but embryogenesis of the CNC and SHF into the aortopulmonary valves and aorta are more complex than merely “filling in their assigned cellular spaces” and deleting unnecessary pharyngeal arteries (Figure 5). These cellular populations have complementary roles in signaling each other, notably, using various proteins—canonical Wnt, non-canonical Wnt, transforming growth factor (TGF)-β, sonic hedgehog (Shh), fibroblast growth factor (Fgf), bone morphogenetic protein (BMP), and Notch—that determine spatial and functional relationships with other cell types during embryogenesis. These pathways also have vital functional roles in adulthood; thus, errors in embryonic signaling that potentially cause outflow tract structural abnormalities such as BAV may also result in AAD in adulthood. An attractive hypothesis is that a lineage-specific cell defect causes the aortic disease that occurs in association with BAV (Jiang et al., 2000; Jiao et al., 2016). In summary, although it has been postulated that the root and tubular phenotypes of AAD may indicate distinct genetic origins (Girdauskas and Borger, 2013), there is still a need for direct supporting evidence (Girdauskas et al., 2011).

## Histopathology of the aorta in TAV and BAV

**Figure 6:**
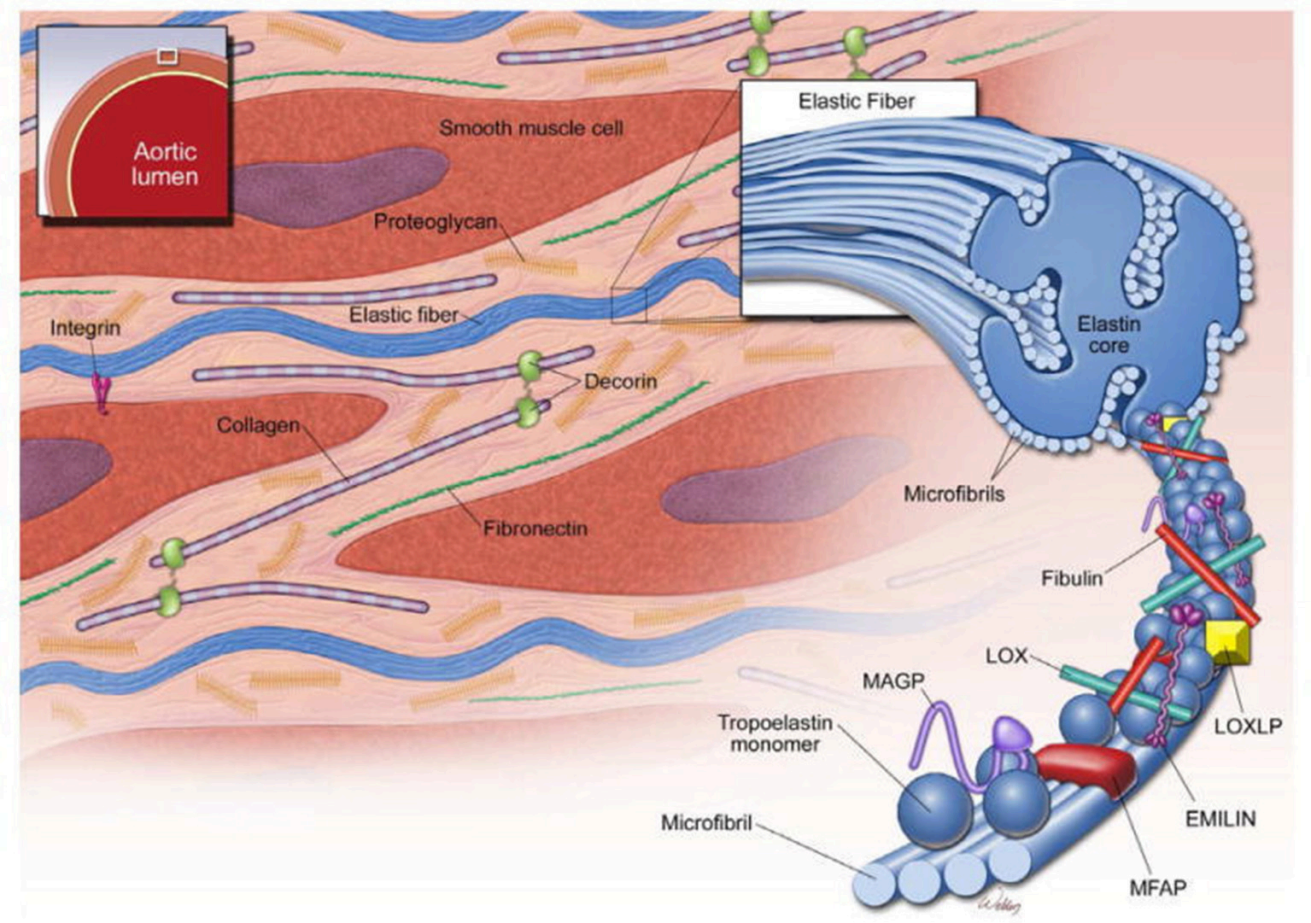
Structure of the ascending aortic media. The media of the aortic wall is composed of vascular smooth muscle cells (SMCs) and an extracellular matrix (ECM) of elastic fibers, collagen fibers, and proteoglycans. Elastic fibers are the major ECM component and provide extensibility to the aortic wall. Cross-linking of tropoelastin monomers by lysyl oxidase (LOX) forms elastin molecules, which in turn cross-link with microfibrils to form elastic fibers. Microfibrils are composed of fibrillin and several microfibril-associated proteins (MFAPs), such as elastin microfibril interface-located protein 1 (EMILIN-1), microfibril-associated glycoproteins (MAGP-1 and -2), and fibulins. Notably, microfibrils provide a substrate for the large latent complex and transforming growth factor-β sequestration. Modified from Wu et al. (2013).

The aorta and large vessels are composed of the intima, a layer of endothelial cells that sit directly on the internal elastic lamina; the media, consisting of concentric layers of smooth muscle cells (SMCs) and their extracellular matrix (ECM); and the adventitia, principally made up of myofibroblasts that produce collagen, able to deal with stresses above physiological pressures (Figure 6). The main mechanical function of the aortic media is providing elastic recoil for pulsatile aortic pressure, enabled by its organized composition of SMCs and ECM.

### Smooth muscle cells

**Figure 7:**
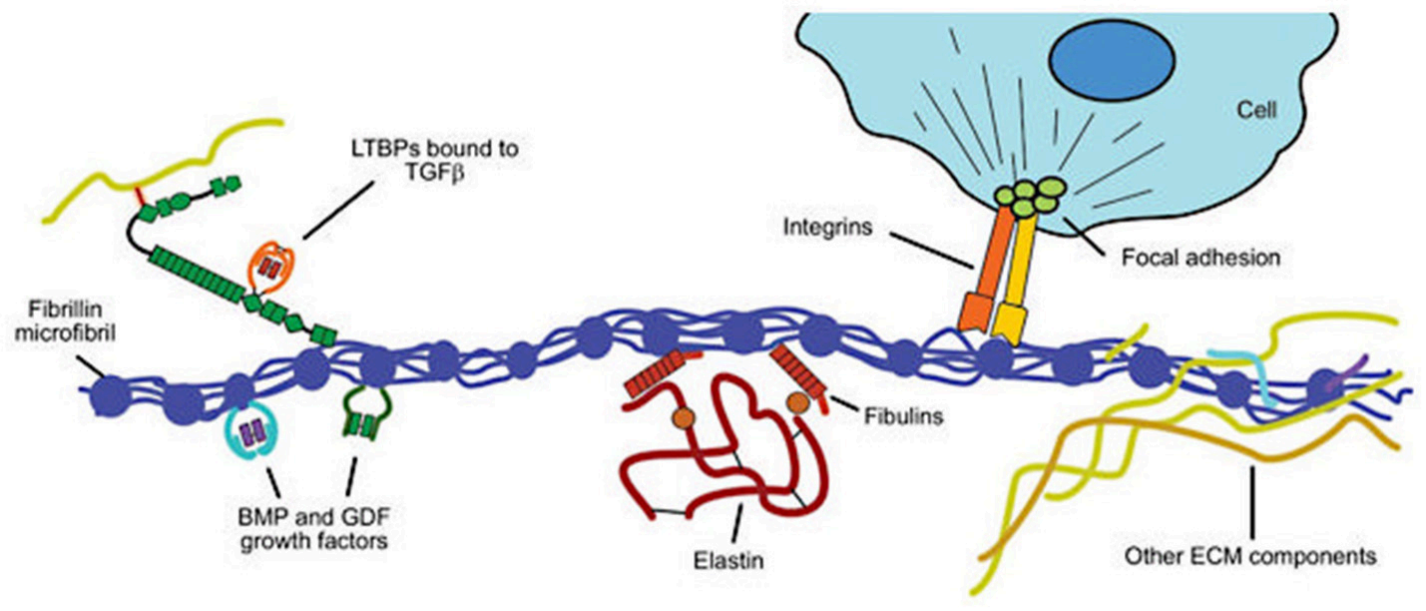
Structural and functional roles of fibrillin in the extracellular matrix. Fibrillin microfibrils associate with elastin to form elastic fibers in the aortic media (see Figure 2). Key functional roles are (i) binding to elastin *via* the fibulins and other extracellular matrix (ECM) glycoproteins; (ii) sequestering transforming growth factor-β (TGF-β) via the large latent complex, bone morphogenetic protein (BMP) and growth and differentiation factors (GDFs); and (iii) linking to smooth muscle cells of the media via integrins. Modified from Robertson et al. (2011).

SMCs are the majority cell type within the aortic wall. They are not terminally differentiated but rather have the ability to express proteins involved in contraction and ECM synthesis during development and in response to mechanical and chemical stimuli (Figure 7). This plasticity, allows transition along a continuum between a fibroblast-like, proliferative, ECM-producing phenotype and a quiescent contractile phenotype. Quiescent contractile SMCs have low production of ECM proteins but express contractile proteins including smooth muscle alpha-actin and myosin heavy chains (Humphrey et al., 2015).

SMCs are bound to elastic fibers, Fbn-1 and collagen type VI, with basal lamina connections linking them to each other and providing a template structure for lamellar (or laminar) organization (Perrucci et al., 2017). Arteries therefore have multiple lamellae (fish scale-like plates) comprising the media, with the number seemingly set during embryogenesis and related to the diameter and stress upon the vessel; thus, the aorta has the greatest number of lamellae. When activated to an immature phenotype, SMCs proliferate and migrate, while producing greater amounts of ECM proteins, thereby regulating the aorta's mechanical properties in response to physiological wall stresses.

At the cell surface, tyrosine kinase, integrin and G-protein receptor-mediated factors (including basic fibroblast, platelet-derived, epidermal, and insulin-like growth factors) favor a proliferative SMC phenotype. Importantly, angiotensin (AT) II mediates both contractile and proliferative phenotypes through its type I and type II receptors, ATR-I and ATR-II, respectively; the former seem to mediate increased TGF-β levels, leading to a proliferative phenotype and ECM remodeling, whereas the latter favor a contractile phenotype.

### Extracellular matrix

The ECM is principally composed of elastin, along with collagen types I, III, IV, V, and VI; fibronectin; Fbn-1; fibulin-4; and proteoglycans of dermatan, chondroitin, and heparin, along with other proteins; these proteins are interspersed with SMCs and form lamellar plates (Wagenseil and Mecham, 2009). The number of lamellae is greater in larger vessels facing greater wall tension and seems to remain stable after birth. Elastic microfibrils are linked to SMCs of adjacent lamellae via integrins σ_5_β_1_ and σvβ_3_, creating an oblique capacitor for vascular stress. Each lamella is oriented obliquely to adjacent lamellae, creating an even distribution of stress across the aortic wall. Apparently, in the normal aorta, SMCs have little active role in managing wall tension and the microfibrillar structure is the major passive contributor.

**Figure 8:**
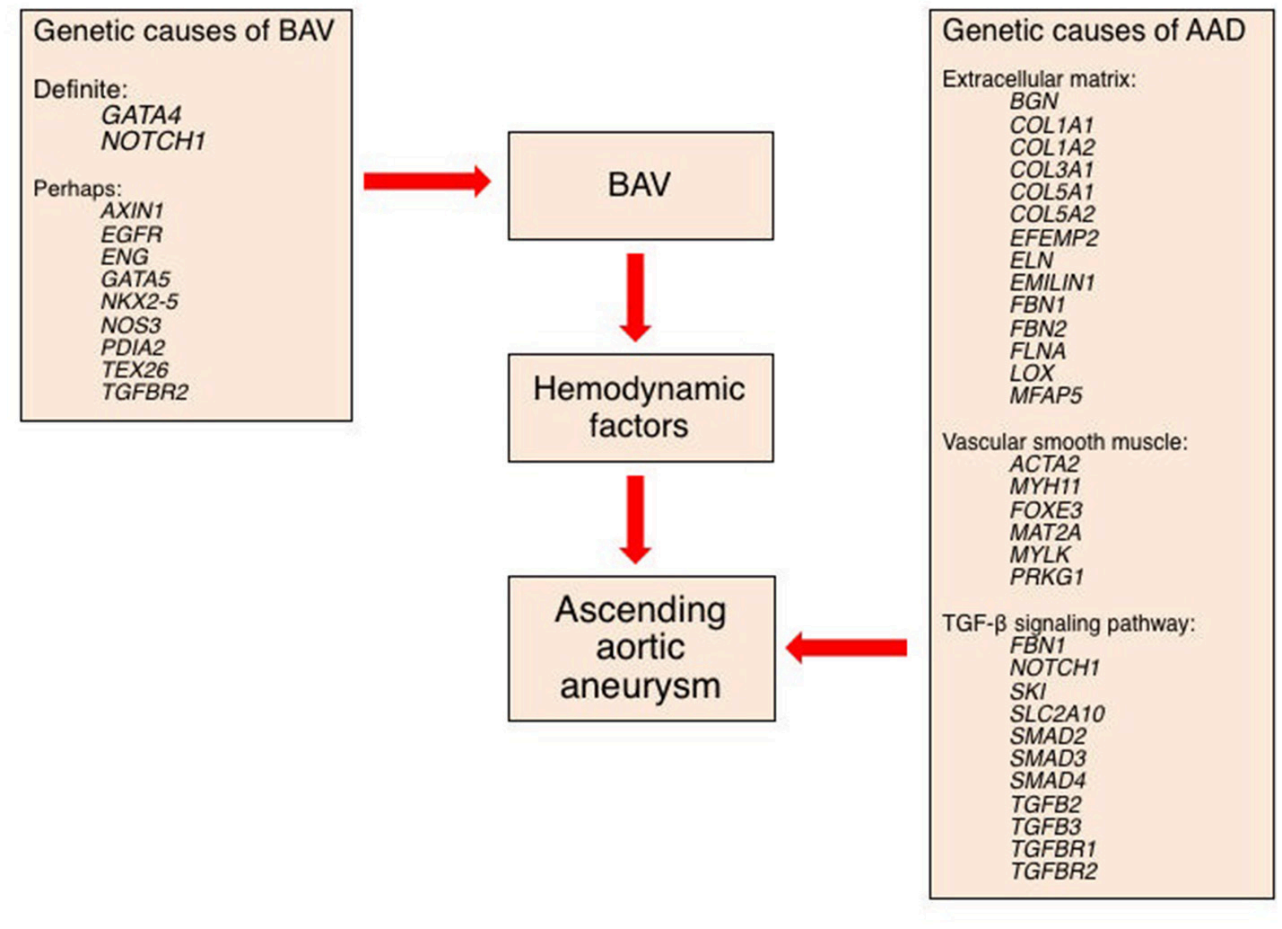
Schematic of a mechanistic approach to the development of thoracic ascending aortic dilation (AAD) ultimately leading to aneurysm. This schematic assumes three groups of AAD etiologic factors: genes causing a bicuspid aortic valve (BAV) that may also be causing AAD; genes causing AAD but not BAV; and hemodynamic factors that contribute to AAD.

Essential to the function of the aortic media, microfibrils provide the structural integrity and organization of the aortic wall, forming a folding, compliant 10–12 nm structure at physiological wall tensions. Structurally, the microfibril is composed of polymeric fibrillin wrapped around an amorphous elastin core, which in turn is formed from monomers of tropoelastin produced by SMCs and covalently cross-linked by lysyl oxidase (Wagenseil and Mecham, 2009; Figure 8). In addition to Fbn-1 and elastin, other proteins including TGF-β binding proteins (LTBP 1–4), emilins, microfibril-associated glycoproteins (MAGP-1 and -2), and members of the fibulin 1–4 family are present in the microfibril (Wu et al., 2013). Fibrillin is notable for its many protein- and integrin-binding sites and its ability to sequester growth factors, notably TGF-β, BMPs and epidermal growth factors (Robertson et al., 2011). In addition to providing a compliant structure, the microfibril serves a cell adhesion function for SMCs, the intima and the adventitia. Collagens I, III, and V are fibril-forming collagens, with types I and III providing high-tensile strength to the vessel wall, in contrast to elastin in the media, which manages physiological tensions.

### TAV vs. BAV aortopathy

AAD unrelated to BAV is characterized by severe elastin degeneration with fibrosis and cystic degeneration of the media in concert with inflammatory histologic changes, along with adventitial and intimal thickening (Balistreri et al., 2013; Forte et al., 2013). However, BAV aortopathy has distinct differences from TAV aortopathy (Table 1); the ascending aorta of patients with BAV generally shows non-inflammatory loss of SMCs, with multifocal apoptosis and medial degeneration (Balistreri et al., 2013) but a fiber architecture similar to that of the normal aorta (Phillippi et al., 2014). Similar to the histologic and molecular perturbations reported in Marfan syndrome, BAV aortic tissues have lower fibrillin content and an increase tissue TGF-β1 levels that is due to the disease (Doyle et al., 2012; Nataatmadja et al., 2013).

**Table 1:** TAV vs. BAV aortopathy.

**Tricuspid aortopathy**	**Bicuspid aortopathy**
**EPIDEMIOLOGY**	
Rare Onset later in life (>70 years) Association with hypertension and other risk factors for aortopathy Lower correlation with severity of aortic stenosis	Frequent in BAV population Onset earlier in life (<70 years) Reduced association with risk factors for aortopathy Moderate correlation with severity of aortic stenosis
**MACROSCOPIC AORTOPATHY**	
Commonly symmetric dilation of the tubular ascending aorta Lower prevalence of aortic stenosis Aortopathy and dissection rarely occurs after AVR	Higher prevalence of aortic root aneurysm Higher prevalence of aortic dilation Higher prevalence of dilation of the outer curve of the ascending aorta Associated with coarctation of the aorta Reported association with type of cusp fusion Common transverse aortic stenosis jet Aortopathy and dissection occasionally occurs after AVR
**MICROSCOPIC AORTOPATHY**	
Severe elastin degeneration Cystic degeneration of the media Inflammatory response often present	Normal fiber architecture Loss of smooth muscle cells with apoptosis Medial degeneration and lower fibrillin content

In the absence of aortic aneurysm, the aorta in BAV often appears macroscopically and histologically normal, or nearly so. But phenotypic variation is seen among patients with BAV undergoing non-aortic surgery, and histologic diversity is evident across different portions of the ascending aorta within the same patient for those with aortic aneurysm. Both the intima and adventitia are thickened (Forte et al., 2013). The convex (outer curve) of the aorta is more frequently dilated in patients with BAV, a change often ascribed to greater shear stress from asymmetric flow across the valve, and has been reported to exhibit greater elastic fiber fragmentation, reduced collagen types I and III expression, and SMC apoptosis (Cotrufo et al., 2005; Della Corte et al., 2008). Matrix metalloproteases (MMP) are also differentially expressed across different aortic sites in BAV, with higher levels of MMP-2 and tissue inhibitor of metalloprotease (TIMP)-3 seen in the concavity of the ascending aorta (Mohamed et al., 2012). These differences are also seen in microRNA expression when comparing convex and concave portions of dilated aortas in BAV (Albinsson et al., 2017). Taken together, these findings strongly support a thesis that active cellular processes are involved in development of bicuspid aortopathy, perhaps or even probably, mediated by hemodynamic forces. But regional histologic findings often do not match expected hemodynamic stresses (Cotrufo et al., 2005; Leone et al., 2012), and many studies have failed to account for the clinical phenotype, notably making little distinction between the dilated aortic root and the dilated tubular aorta.

## Hemodynamic and genetic mechanisms of BAV-related aortopathy

Much of what we believe about the etiology of AAD and thoracic aortic dissection observed in association with BAV is based on two competing, or more likely complementary, etiologic hypotheses of increased wall stress and pathologic structural or functional deficits of the aortic wall. Yet, the evidence for each is neither strong nor specific to BAV-related aortopathy. Much is derived from aortopathy observed in association with TAV and comes exclusively from end-stage disease tissue obtained at surgery because tissue is rarely obtained earlier in disease, when aortic dimensions are smaller. This is understandable but limits insight into mechanisms that may be specific to BAV or aortic phenotype, and biological mechanisms of early-stage aortopathy.

### Hemodynamic mechanisms of BAV-related aortopathy

**Figure 9:**
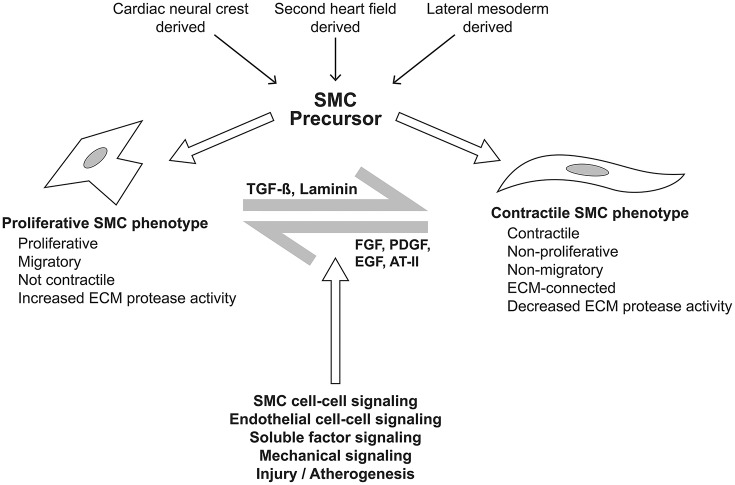
Plasticity of smooth muscle cell phenotype. The spectrum of smooth muscle cell (SMC) phenotype from proliferative to contractile phenotypes is dependent upon signaling from multiple sources. The sources include adjacent SMCs and vascular endothelial cells, mediated by cell-cell interaction and soluble factors including transforming growth factor-β (TGF-β), laminin, fibroblast growth factor, platelet derived growth factor, epidermal growth factor, and angiotensin II, amongst others. Mechanical signaling *via* the extracellular matrix, cell-cell mechanical sensing, and the intracellular cytoskeleton and primary cilium are propagated through the intracellular dense plaques. These signals yield changes in cytoskeletal architecture and drive plasticity across the spectrum of SMC phenotype.

There is extensive evidence that blood flow and shear stress in the tubular ascending aorta are markedly altered by a BAV, even in the absence of significant flow obstruction (Entezari et al., 2014; Garcia et al., 2016; Cao et al., 2017; Raghav et al., 2017; Shan et al., 2017; Figure 9). However, fused-leaflet morphology is not a sole determinant of helicity of blood flow or region of maximal wall stress in the tubular ascending aorta (Raghav et al., 2017; Shan et al., 2017), as age and clinical characteristics are important (Burris et al., 2016; Girdauskas et al., 2016b; van Ooij et al., 2016; Shan et al., 2017).

Abnormal flow characteristics from the BAV impose abnormal mechanical stresses upon portions of the aortic wall, leading to alterations of cell-mediated processes (Atkins and Sucosky, 2014; Shan et al., 2017). In general, these processes include dysregulation of ECM and medial elastin fiber degeneration (Guzzardi et al., 2015), at least in part mediated by MMP-dependent pathways. Comparing regions of the tubular ascending aorta having 4-dimensional flow MRI-assessed high vs. normal wall shear stress, the former areas exhibit thinner elastin fibers and less total elastin content, indicating increased medial elastin degradation (Guzzardi et al., 2015). Likewise, evidence of ECM dysregulation has been found in regions of high wall shear stress in the form of increased expression of *TGFB1*; *MMP* types 1, 3 and perhaps 2; and *TIMP1*. However, there is marked inter-individual variation, implying that the pathways may be important as a response mechanism for aortic wall shear stress that leads to reduced elasticity of the aorta, although their value as a prediction tool is limited. Importantly, they may also merely reflect end-stage disease, with mechanosensors in the aortic wall initiating earlier transcriptional and post-transcriptional pathways mediated by microRNA expression (Albinsson et al., 2017). There are several mechanisms of mechanotransduction, including the endothelial glycocalyx layer on the luminal surface of the aorta, basal integrins, primary cilia, and platelet endothelial adhesion molecule-1 (Russell-Puleri et al., 2017), amongst others. Notably, the complex of polycystin-1 (derived from the *PKD1* gene) and polycystin-2 forms a mechanosensitive cation channel of the primary cilia, which serve as mechanosensors by interacting with filament-A bound to cytoskeletal actin (Patel and Honore, 2010).

Direct comparison of multiple murine elastase and genetic aortic aneurysm models has provided useful insights (Bellini et al., 2017). Aneurysmal development was found to correlate with increased wall stiffness as distensibility was lost, as perhaps adventitial collagen became the principal controller of wall stiffness. The inability of intramural SMCs and myofibroblasts to maintain nearly normal circumferential wall stiffness in these models is consistent with a compromised ability to mechanoregulate the ECM. Mechanistically, SMCs and myofibroblasts secrete TGF-β1, platelet-derived growth factor, and AT II, amongst other factors that regulate their own function and cell fate, as well as that of nearby cells, creating a local environment sensitive to local hemodynamic forces.

The absence thus far of identified variants in genes coding for mechanosensing proteins that play a human AAD implies either that these variants are embryonic lethal or that mechanosensing is not a primary lesion in AAD, despite views otherwise (Humphrey et al., 2014, 2015). However, downstream effectors have a comprehensive role in AAD, especially proteins involved in generation and maintenance of the ECM, vascular SMC contraction or metabolism, and mediation by the TGF-β signaling pathway.

### Genetic mechanisms of BAV-related aortopathy

It is reasonable to hypothesize several possible genetic mechanisms of increased risk of BAV-AAD or TAV-AAD (Figure 9). These likely include (i) one or more genes responsible for BAV that also are mechanistically responsible for AAD (pleiotropy); (ii) the independent actions of one or more genes upon a phenotype such as BAV (polygenic influence), while other genes act upon the AAD phenotype; (iii) two or more genes that mechanistically interact to produce a single phenotype (epistasis); and (iv) genetic variants that interact with hemodynamic factors to produce AAD (gene-by-environment interaction). Given the complexity of the disease, clinical factors that alter disease progression, the mixed inheritance pattern and association of numerous genes with AAD in animal models and the general population, it is reasonable to investigate individual and combined contributions of all four mechanisms.

Numerically, few cases of BAV-AAD are associated with extra-cardiac manifestations. A small proportion of patients with BAV have coarctation of the aorta, while an even smaller proportion have Turner, Marfan, Loeys-Dietz, and other even rarer syndromes resulting from *FBN1, COL3A1, SMAD3*, and *TGFBR1/2* genetic variants, amongst others. The majority of AAD in both TAV and BAV is non-syndromic, although at least a third of patients have identified genetic variants for familial AAD including variants of *ACTA2, MYH11, MYLK, FBN1*, and *TGFB2*, amongst others. These imperfect associations are noteworthy in that they emphasize the complexity of embryonic development, the adult onset of genetic AAD disease, and, for syndromic AAD, the importance of specific proteins across a wide variety of organ systems. The other lesson is that variants in or near a single gene lead to a spectrum of severity and manifestations of AAD, akin to pleiotropy; a notable example is *FBN1* variants, for which clinical phenotype can range from severe forms of Marfan syndrome to merely an increased risk of AAD in adulthood.

Human BAV has been associated with rare, but highly penetrant, exonal variants in *NOTCH1* (Garg et al., 2005; Mohamed et al., 2006; Foffa et al., 2013; Dargis et al., 2016), *AXIN1* (Wooten et al., 2010), *EGFR* (Dargis et al., 2016), *ENG* (Wooten et al., 2010), *GATA5* (Padang et al., 2012; Bonachea et al., 2014a; Shi et al., 2014), *NKX2*–5 (Qu et al., 2014; Dargis et al., 2016), *NOS3* (Girdauskas et al., 2017), *PDIA2* (Wooten et al., 2010), *TEX26* (Dargis et al., 2016), and *TGFBR2* (Dargis et al., 2016). Furthermore, BAV presenting together with AAD has been associated with rare variants in *NOTCH1* (McKellar et al., 2007; Girdauskas et al., 2017), *AXIN1* (Girdauskas et al., 2017), *TGFBR2* (Martin et al., 2011; Girdauskas et al., 2017), *FBN1* (Pepe et al., 2014), *SMAD2* (Prapa et al., 2014), *NOS3* (Girdauskas et al., 2017), *ACTA2* (Guo et al., 2007), *TGFB2* (Lindsay et al., 2012), and other genes (Girdauskas et al., 2017). To date, the only locus containing common variants associated with BAV is *GATA4* (Yang et al., 2017), but this locus has not been associated with AAD. More than 40 genes have been linked to BAV in mice or hamsters (Wu et al., 2017).

Approximately 30 genes are associated with AAD in the general population, but many of these are rare exonal variants identified in only a few families or individuals (Brownstein et al., 2017). The majority encode proteins involved in generation and maintenance of the ECM *(BGN, COL1A1, COL1A2, COL3A1, COL5A1, COL5A2, EFEMP2, ELN, EMILIN1, FBN1, FBN2, FLNA, LOX, MFAP5*); vascular SMC contraction or metabolism (*ACTA2, MYH11, FOXE3, MAT2A, MYLK, PRKG1*); or TGF-β signaling (*FBN1, NOTCH1, SKI, SLC2A10, SMAD2, SMAD3, SMAD4, TGFB2, TGFB3, TGFBR1, TGFBR2*). Almost one-quarter of patients with AAD possess a mutation in one of these genes. Rare or uncommon mutations in six of these genes [*AXIN1* (Girdauskas et al., 2017), *ELN* (Girdauskas et al., 2017), *FN1* (Girdauskas et al., 2017), *NOS3* (Girdauskas et al., 2017), *NOTCH1* (Garg et al., 2005; McKellar et al., 2007; Foffa et al., 2013; Bonachea et al., 2014b; Kerstjens-Frederikse et al., 2016) and *TGFBR2* (Dargis et al., 2016)] have been associated with AAD in patients with BAV. Thus, it is unlikely that these genes are responsible for a significant proportion of the AAD seen in this valvular disorder. However, *FBN1* has been shown to have common variants that are associated with AAD in patients with BAV, and it is therefore a leading mechanistic candidate, especially given its dual structural and TGF-β signaling roles (LeMaire et al., 2011; Pepe et al., 2014; Guo et al., 2016; Girdauskas et al., 2017).

## Integrated mechanism(s) of BAV aneurysm and dissection

Over the last decade of genetic studies that have identified highly penetrant coding variants in a set of interrelated genes that are associated with aortic disease, it has become apparent that the ECM and SMCs are the important factors in aortic integrity and function. The predominance of identified genetic causes of AAD coming from ECM, vascular SMC contraction or metabolism, or the TGF-β signaling pathway points to avenues for investigating aortopathy and allows prioritization of candidate pathways, especially for a role of common variants in *FBN1*.

### Why bicuspid aortopathy may be two or more diseases

As previously mentioned, there is reasonable evidence that AAD phenotypes mirror distribution of embryonic SHF and CNC cells. Clinical similarity of the BAV aortic root phenotype to the aortic aneurysm phenotype of Marfan syndrome further implies common mechanisms and cellular pathways. This similarity is reinforced by the observation of decreased tissue fibrillin and increased TGF-β content in both (Nataatmadja et al., 2013). But surprisingly, if we expected the root aneurysm phenotype of both Marfan syndrome and BAV to mirror the distribution of CNC migration, BAV morphotype is not well-correlated with aortic aneurysm morphotype (Jackson et al., 2011; Habchi et al., 2017).

Characteristics of tubular AAD differ between bicuspid and tricuspid aortopathy. There is extensive evidence that the hemodynamic forces on the aortic wall seen in both non-stenotic and stenotic BAV differ between the vessel's concave and convex aspects and also differ from those seen in the non-stenotic TAV (Shan et al., 2017). These differences probably, but not certainly, drive the difference in gross morphology and histopathology across the aorta's axial plane. Further, numerous studies have identified biochemical differences between BAV and TAV that drive, or occur in response to, histopathological differences. For BAV, these include SMC apoptosis and increased MMP secretion, whereas for TAV, these include elastic fiber fragmentation, cystic medial necrosis and fibrosis (Boyum et al., 2004; Balistreri et al., 2013).

If we postulate a genetic background to BAV aortopathy, it would be reasonable to assume that *FBN1* variants would dominate the genetic cause of aortic root aneurysm, as common *FBN1* variants have been associated with non-syndromic bicuspid aortic aneurysm, independent to the position of the aneurysm (LeMaire et al., 2011). This has not been shown, however; deleterious variants in *NOTCH1, AXIN1* and *NOS3* have been found to be more common than deleterious *FBN1* variants, at least in one cohort (Girdauskas et al., 2017). However, common *FBN1* variants have not yet been associated with non-syndromic bicuspid aortic aneurysm, without reference to the position of the aneurysm (LeMaire et al., 2011). To date, *FBN1* variants have not been associated with BAV in the absence of aortopathy.

For tubular AAD, it is reasonable to postulate that hemodynamic factors including predominant jet direction, along with several genetic factors may have a role in bicuspid aortopathy. It could also be argued that the genetic variant associated with BAV, especially the *NOTCH1* variant, might also be causal for aortopathy, but supporting data are lacking at this time.

### Fbn-1 and the TGF-β pathway

Fbn-1 is a backbone microfibrillar protein with structural and signaling functions. When abnormal Fbn-1 was first identified as the cause of Marfan syndrome in 1991 (Dietz et al., 1991), it was believed that the coding mutations resulted in a structural weakness of microfibrils (Matt et al., 2008), leading to arterial wall “weakness” and the syndrome's vascular manifestations. Supporting this assertion, more severe or earlier aortic disease had been associated with *FBN1* truncating or splicing variants (Schrijver et al., 2002; Baudhuin et al., 2015) and with variants in exons 24–32 (Faivre et al., 2007). But this is a vastly simplistic overview.

The ~3,000 currently identified phenotypically important *FBN1* mutations fall into two classes. The more common class of dominant mutations (with a single coding variant on one chromosomal copy of *FBN1*) results in a mixture of mutated and normal, non-mutated Fbn-1 protein in the ECM, a pattern called a dominant negative (DN) mutation. The result is aberrant Fbn-1 folding, which in turn leads to a disorganized ECM and weakened microfibrillar structure. Alternatively, the less common class of haploinsufficiency (decreased protein production from the mutated *FBN1* gene on one, or rarely two, chromosomes) results in decreased amounts of normal Fbn-1 protein present in the ECM; compared with DN mutations, this class of defects carries greater risk of aortic aneurysm or vascular dissection (Franken et al., 2017). To date, it is not readily apparent whether the DN mutation class or haploinsufficiency class of *FBN1* variants, or their combination, plays an important role in BAV aortopathy.

**Figure 10:**
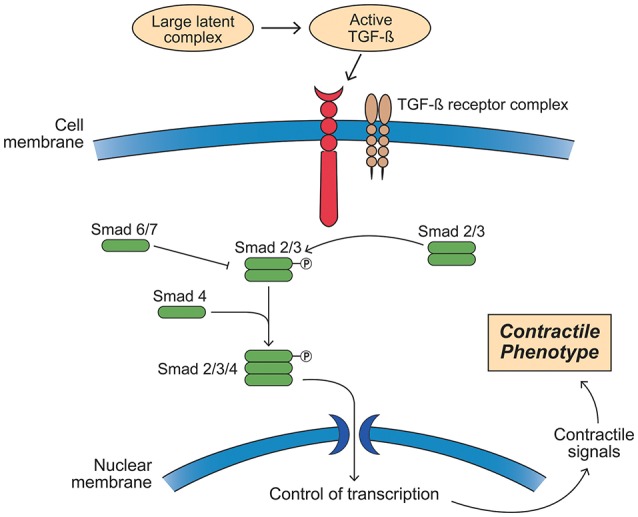
Activation of the transforming growth factor-β (TGF-β) signaling pathway leading to a smooth muscle cell contractile phenotype. Members of the TGF-β superfamily that include TGF-βs, bone morphogenetic proteins (BMPs), and growth and differentiation factors (GDFs) have similar functional properties regulating cell growth, differentiation, apoptosis, and extracellular matrix synthesis in vascular smooth muscle cells (SMCs). TGF-β ligands are synthesized as latent precursor molecules (LTGF-β), which are activated via proteolytic cleavage. Active TGF-β signaling is transmitted through two types of transmembrane serine/threonine protein kinase receptors: TGF-β type I (TGFβRI) and principally type II (TGFβRII) and mediated by a sequence of phosphorylated Smad proteins. In addition to the canonical Smad signaling pathway that directly regulates the transcription of Smad-dependent target genes, TGF-β function can also be mediated by Smad-independent pathways including MAPK signaling pathways, such as p38 MAPK and c-Jun NH2-terminal kinase, phosphatidylinositol 3-kinase/Akt pathway, and Wnt signaling. TGF-β signaling via TGFβRII plays a pivotal role in both second heart field and cardiac neural crest derived SMC phenotype differentiation during vascular development as well as SMC phenotypic switching in disease states. TGF-β signaling induces SMCs to change shape into elongated SMC shape accompanied by an up-regulation of SMC contractile proteins.

Fbn-1's principal signaling functions are mediated by TGF-β1, BMP, and epidermal growth factor. Both mutated and decreased Fbn-1 protein lead to impaired ability to sequester TGF-β1 on the latent TGF-β binding protein, resulting in increased tissue and circulating levels of this growth factor (Mathieu et al., 2015). TGF-β1 is activated by proteolytic cleavage from its inactive form on the latent TGF-β binding protein, a process that is governed by both wall stress and integrin activation by MMP and other mechanisms (Wipff and Hinz, 2008; Forte et al., 2016; Perrucci et al., 2017). Once released, TGF-β1 binds to cell surface TGF-β1 receptor complexes (TGFRB1/2). This process in turn activates the canonical Smad 2/3 and the Smad 1/5/8 transcription factors by phosphorylation, leading to a contractile SMC phenotype under normal developmental circumstances (Figure 10). In contrast, TGF-β1 mediated non-canonical Smad-independent pathways can induce increased MMP activity and ECM degradation. Complicating this relationship, AT II may also be able to activate the Smad2 pathway either indirectly by increasing tissue TGF-β1 or directly via activation of the ATR-I (Nagashima et al., 2001; Franken et al., 2015). Furthermore, emerging evidence suggests that there may be epigenetic control of TGF-β1 in aortic aneurysm and vascular disease that adds yet an additional layer to this already complex picture (Leeper et al., 2011; Shah et al., 2015; Forte et al., 2016). Thus, TGF-β1 has important roles in tissue fibrosis, cellular differentiation and proliferation, and ECM remodeling through several pathways (Forte et al., 2010).

The strength of the association between *FBN1* variants and aortic disease in BAV is similar to that in Marfan syndrome. LeMaire and colleagues have identified a ~250-kbp locus in *FBN1* that is associated with thoracic aortic aneurysm and dissection in both patients with non-bicuspid and patients with BAVs (LeMaire et al., 2011). This is an important finding as it provides the first identification of common variant(s) in a gene having a role in aneurysmal disease in Marfan syndrome, in the general population and in BAV, for which a pathogenic mechanism had already been established.

Manipulation of TGF-β1 function is an attractive therapeutic approach implemented in several high-quality, relatively short-term clinical trials of the angiotensin receptor blocker (ARB) losartan with promising, but not definitive results. The distinction between qualitative and quantitative *FBN1* mutations is important as this drug decreases TGF-β1 production, offsetting the increased circulating levels of this growth factor seen in Marfan syndrome and BAV (Nataatmadja et al., 2013). In a sub-study of the COMPARE trial examining the effect of losartan upon on-going aortic root dilation in patients with Marfan syndrome, the drug was more effective in slowing progression in patients with haploinsufficient variants than in those with protein mutational variants, implying that Fbn-1's role in modifying TGF-β1 is an important mechanism of aortic root dilation (Franken et al., 2017). This finding does not necessarily imply that structural weakness of microfibrils is unimportant, however.

### NOTCH1

Rare but highly penetrant *NOTCH1* variants in two syndromic pedigrees (Garg et al., 2005) were the first definitive genetic associations with BAV and have been further identified in specific BAV root aneurysm and other phenotypes (McKellar et al., 2007; Dargis et al., 2016; Girdauskas et al., 2017). In addition, rare *NOTCH1* variants have been associated with left and right heart structural lesions and with other, non-cardiac phenotypes (Luxan et al., 2013). Notch 1–4 proteins are heterodimeric transmembrane receptors for Jag1/2 and Dll1/3/4, and they promote endothelial-to-mesenchymal transformation of SHF cells to form the outflow tract valves. The strongest evidence thus far for a role for Notch proteins in AAD is the presence of proximal aortic disease in Notch1^+/−^; NOS3^−/−^ mice (Koenig et al., 2015) and a possible association with decreased endothelial-to-mesenchymal transition in human BAV endothelium (Kostina et al., 2016). Although, Notch1 is essential for outflow tract development and may contribute to development of some BAV, its role in thoracic aortic aneurysm, if any, is uncertain.

### GATA4

The important role of the GATA sequence binding proteins in embryonic cardiac development is well-recognized (Martin et al., 2015) and is reinforced by presence of BAV in mouse *GATA* knockouts (Laforest and Nemer, 2011; Laforest et al., 2011), by association of uncommon variants with BAV (Bonachea et al., 2014a; Shi et al., 2014), and recently by identification of common *GATA4* variants in human BAV (Yang et al., 2017). However, although some patients with BAV having GATA variants were found to have thoracic aortic disease, there is currently no evidence that these variants play a role in AAD.

## Is there a biomarker for aortic dissection?

There may be some value to having a circulating biomarker that can identify future aortic aneurysm in young people in order to direct anti-hypertensive or other preventive therapy for AAD. Perhaps value also could come from identifying a highly *predictive* risk index of aortic dissection in a cohort at perceived higher risk, such as patients with aortic aneurysm. The biomarker would need to predict risk over a reasonable length of time between pragmatic assessments. Aortic dimension has been the most used marker of aortic dissection risk, yet it provides little prognostic information over the clinically important range of 40–55 mm (2010 ACCF/AHA/AATS/ACR/ASA/SCA/SCAI/SIR/STS/SVM Guidelines For the Diagnosis and Management of Patients with Thoracic Aortic Disease Representative, Erbel et al., 2014; 2010 ACCF/AHA/AATS/ACR/ASA/SCA/SCAI/SIR/STS/SVM Guidelines For the Diagnosis and Management of Patients with Thoracic Aortic Disease Representative Members et al., 2016), while the majority of aortic dissections occur at dimensions less than these (Pape et al., 2007). This limitation has led to other approaches such as examining morphometry (Biaggi et al., 2009; Habchi et al., 2017), hemodynamic factors (Atkins et al., 2016; DeCampli, 2017; Raghav et al., 2017), the position and shape of the aneurysm (Schaefer et al., 2008; Della Corte et al., 2014b), aortic growth rate (Elefteriades and Farkas, 2010), and aortic distensibility parameters (Benedik et al., 2013). To date, observed population-based associations are weak and are unlikely to provide any predictive value for the individual patient.

However, as we learn more about aortic disease, it is possible that circulating tissue markers will reflect the biology of the aortic wall. Overall, the fundamental weakness of this approach is that an aortic dissection signal could be diluted by other, much larger tissue signals. In addition, because dissection is likely a phenomenon having varied risk factors (hemodynamic, genetic, aortic size, and others), it would be reasonable to assume that multiple markers would be required. Based on known biology, candidate biomarkers can potentially include (i) structural proteins of the smooth muscle contractile network such as actin, myosin, and fibrillin; (ii) regulatory and structural proteins of the aortic ECM such as collagen, elastin, MMPs and their inhibitors; and (iii) ligands and receptors of the TGF-β pathway.

### MMPs and their inhibitors

MMPs, notably MMP-1, 2, 8, and 9, control degradation of the ECM and other cell processes. Tissue and circulating levels of MMP-2 and 9 are the best examined and have been reported to be elevated (Koullias et al., 2004; Schmoker et al., 2007) or not (Tscheuschler et al., 2016; Wang et al., 2016) in patients with thoracic aortic aneurysm, and higher in patients with BAV than in patients with TAV (Boyum et al., 2004; LeMaire et al., 2005; Ikonomidis et al., 2012; Wang et al., 2016). On their own, these data don't provide a complete picture as TIMPs modify the actions of MMPs. Circulating and tissue TIMP levels have been shown to be elevated (Mohamed et al., 2012) or not (Boyum et al., 2004; LeMaire et al., 2005; Ikonomidis et al., 2013; Wang et al., 2016) in patients with BAV and in patients with a thoracic aortic aneurysm. It is very likely that circulating levels of MMPs and TIMPs do not accurately reflect aortic wall tissue levels, comparable to the case for other biomarkers, and therefore do not have a prognostic role in aortic dissection, especially over the range of aortic diseases.

### Transforming growth factor-β

TGF-β has a well-established causal role in the vascular complications of Marfan syndrome (Matt et al., 2008; Hillebrand et al., 2014). Variants in its receptors *TGFBR1, TGFBR2, TGFB2, and TGFB3* are responsible for Loeys-Dietz syndrome types 1, 2, 4, and 5, respectively, along with arterial tortuosity, thoracic aortic aneurysm and BAV disease (Brownstein et al., 2017). As a biomarker, circulating TGF-β1 is elevated in patients with Marfan syndrome, NOTCH1-associated aneurysm and BAV-associated aneurysm (Hillebrand et al., 2014). In seminal work, Forte and colleagues examined mRNA expression of a range of TGF-β pathway genes (*TGF-*β*1, MMP-2/14, ENG*, and others) from non-dilated and dilated aortas of patients with BAV, identifying complex relationships between gene and protein expression in the aorta, and aortic size (Forte et al., 2017). *TGF-*β*1* mRNA was elevated in non-dilated aortas but less so in dilated ones. In contrast, serum levels of TGF-β1 were lower in non-dilated aortas and not significantly elevated in dilated ones. These conflicting data underscore the need for further investigation.

To date, there is no circulating biomarker that can yet provide prospective information for either aortic aneurysm or dissection. There is also no reasonable and safe method for physical biopsy of the ascending aorta. A pragmatic approach of allowing targeted drug or other therapy for a high-risk cohort to prevent an event some 10–60 years in the future may include assessment of genetic markers, along with conventional and molecular imaging.

## Conclusion

AAD has been ascribed to both the hemodynamic consequences of normal and abnormal BAV morphology and to the effect of rare and common genetic variation upon function of the ascending aortic media. We propose an overall thesis that compared with tubular AAD, aortic root AAD has a stronger genetic etiology, perhaps related to identified common non-coding *FBN1* variants that are associated with AAD in patients with tricuspid and BAVs. In patients with BAV having tubular AAD, we propose a stronger hemodynamic influence, but one that is still based on a functional deficit of the aortic media of genetic or epigenetic etiology. The pathogenesis of AAD likely involves both structural coding variants and non-coding variants, and thus far has not been related to identified genetic etiologies of BAV, notably, variants in *NOTCH1* and *GATA4*.

## Author contributions

All the authors substantially contributed to (1) the conception or design of the work, acquisition a revision of literature data; (2) drafting the work or revising it critically for important intellectual content; (3) final approval of the version to be published; and All authors agree to be accountable for all aspects of the work in ensuring that questions related to the accuracy or integrity of any part of the work are appropriately investigated and resolved.

### Conflict of interest statement

The authors declare that the research was conducted in the absence of any commercial or financial relationships that could be construed as a potential conflict of interest.
